# Experience of using a smartphone WeChat applet for dental anxiety assessment and preoperative evaluation: A nationwide multicenter study

**DOI:** 10.3389/fpubh.2022.900899

**Published:** 2022-07-18

**Authors:** Xilu Huang, Jie Zeng, Nan Zhao, Lin Fan, Dijiao Ruan, Jing Wang, Xiaomei Hong, Cong Yu

**Affiliations:** ^1^Department of Anesthesiology, Stomatological Hospital of Chongqing Medical University, Chongqing, China; ^2^Chongqing Key Laboratory of Oral Diseases and Biomedical Sciences, Chongqing, China; ^3^Chongqing Municipal Key Laboratory of Oral Biomedical Engineering of Higher Education, Chongqing, China; ^4^Engineering Research Center of Fujian University for Stomatological Biomaterials, Xiamen Medical College, Xiamen, China

**Keywords:** intelligent personality assessment, WeChat applet, dental treatment, dental anxiety, comfort therapy

## Abstract

**Introduction:**

Dental anxiety is a multivariate phenomenon that regularly occurs during a dental procedure. Although it may lead to patients' safety concerns and adverse events in routine treatment, it is often ignored. The purpose of this research is to develop a novel WeChat Applet for dental anxiety (WADA) with the following features and aims: (1) to help patients with dental anxiety management; (2) provide patient with a physical status self-evaluation; and(3) provide a platform for online assessment and tele-consultancy by dentists. We aimed to test and verify whether such an applet could play a beneficial role before and after a dental procedure and facilitate management of high-risk patients during the COVID-19 pandemic.

**Materials and methods:**

During the 12-month survey period (August 2020 to July 2021), a total of 180 patients aged 3–74 years from eight different cities (*n* = 180 at the end of treatment, *n* = 25 for the System Usability Scale (SUS) and follow-up interview) and 20 medical staff from eight different cities (*n* =20 for follow-up interview) were evaluated by WADA. At the end of the survey period, the results of the interviews were analyzed thematically.

**Results:**

WADA assessment results from 180 patients and follow-up interview results from 45 participants were analyzed. In this study with a male to female ratio of 2:3, 75% were found to be suffering from dental anxiety, 86% were found with postoperative complications, and 11 cases were found to have contraindications to surgery. The total SUS score for WADA is 72.25 above the mean score, proving that WADA is a relevant and useful tool before and after dental treatment. Based on the results of the interviews, the following themes were identified: patient satisfaction; dentists' effectiveness; multi-center data integration; and increase its frequency of usage.

**Conclusions:**

The WADA was developed for dental procedures and is effective for reducing treatment risks, improving patients' satisfaction and dentists' convenience, especially in terms of facilitating management of high-risk patient during the COVID-19 pandemic.

## Introduction

Despite the recent innovations and technological advances in modern dentistry, dental anxiety continues to be widespread (1, 2). It occurs globally and is considered a public health problem (3). Dental anxiety disorder is a complex phenomenon related to a variety of factors (4), and according to study with incomplete statistics, 83.1% of Chinese adult patients had moderate to high dental anxiety and 16.2% met the criteria of dental phobia (Modified Dental Anxiety Scale [MDAS] score ≥ 19) (5). People suffering from dental anxiety often try to avoid or delay dental treatment, leading to deterioration of oral health and a reduced quality of life. The progression of untreated oral infections, combined with feelings of remorse, humiliation, or worthlessness, contributes to an increase in dental anxiety, and the vicious cycle continues. This has been described by several researchers as a “dynamic vicious cycle”(6). Identifying and preventing dental anxiety in the early stages of oral disease is considered a key approach to improving patients' oral health and dental-visit experience (7).

eHealth (electronic health) is a broad term that describes the use of electronic devices to provide healthcare. Mobile health (mHealth) is a component of eHealth and involves the use of mobile devices to collect data about an individual's health status and provide information to professionals and patients in real-time (8, 9). The market for mHealth applications has exploded in the last decade (10). There is a wide range of medical applications available at home and abroad, such as MedActionPlan Pro (MPP), Kræftværket, and iManage, among others (11–13). The scale of mobile internet continues to expand, with data showing that as of September 30, 2020, the number of global Internet users was nearly 4,929,926,187. More than half (2,555,636,255; 51.8%) of these Internet users are in Asia (14). In 2017, WeChat applets were launched, which were downloaded by 1.09 billion users, and WeChat-based small programs are used by 400 million users every day (15). It is expected that such an applet will be the main form of mobile Internet applications and a promising way to increase the frequency of application use, including that for health management and self-monitoring, which is already popular.

The WeChat Applet for dental anxiety (WADA) was initially developed by the Comfort Dentistry Center of the Stomatology Hospital affiliated with the Chongqing Medical University in 2018. Today, with the continuous progress of artificial intelligence technology, the development of an intelligent preoperative evaluation platform system combined with artificial intelligence can partially solve the problems of high information load and high repetitive labor intensity of anesthesiologists in the evaluation of dental treatment, and this method is less prone to errors and omissions. Therefore, the idea of creating a WeChat-based applet to manage patients more conveniently and efficiently was discussed by the doctors of Comfort Dentistry. It was proposed that a remote and intelligent medical assistance platform system needed to be built with the integration of information previously obtained by the Comfort Dentistry Center.

The purpose of this research was to develop a novel applet, WADA, and to investigate how patients seeking dental treatment evaluate WADA after use. We aimed to test and verify whether such an applet could play a beneficial role before and after a dental procedure and facilitate management of high-risk patients during the COVID-19 pandemic.

## Methods and analysis

### Study design

A participatory design was adopted (16). It is a design that aims to actively involve all stakeholders in the design process to help ensure that the results meet their requirements and improve usability. Participatory design is guided by a fundamental ethical stance that end users who may be affected by the design in the future should have a voice in the process (17, 18). Face-to-face participatory design sessions were conducted, which is a way for end users to actively co-design technical solutions together with researchers and product designers, involving patient representatives, anesthesiologists, dentists, and computer scientists. The aim was to make WADA more attractive and effective for all users. Additional face-to-face and on-line participatory sessions were planned during the study when necessary.

### Participants and recruitment

Participants for the 12-month WADA survey were recruited by Comfort Dentistry or anesthesia departments in dental offices nationwide. The inclusion criteria were as follows: ability to access the Internet *via* cellular data or Wi-Fi with smartphones either independently or with the help of relatives. Medical staff who were involved in the WADA co-creation development process and patients who were unable to use their smartphones to complete the questionnaire were excluded. Two hundred participants, including 180 patients and 20 medical staff (Table 1), were recruited during the 12-month test period (August 2020 to July 2021) and 180 patients all used WADA before and after treatment. One hundred and eighty patients completed their dental treatment. At the end of the test period, the study team used a stratified random sampling method to select 30 patients for analysis using the System Usability Scale (SUS) and follow-up interview as well as 20 medical staff for the follow-up interview. Of the 30 patients, three were unable to complete the interview for personal reasons, one refused to be interviewed, and one patient passed away due to illness. These patients were excluded, and a total of 45 participants (25 patients and 20 medical staff) were included in the follow-up interviews and randomly mixed in terms of region, sex, and age. The study was conducted during the August 2020 to July 2021 pandemic. First, for protection against COVID-19, the patients were required to present the results of a nucleic acid test within 48 h and a trip code and health code (a way to ensure that you are not in contact with an infected person) before being granted access into Stomatology Hospital affiliated with the Chongqing Medical University. Second, the health care workers took precautions (such as wearing protective clothing, masks, and face masks) and underwent nucleic acid tests once a week to ensure that they were not infected with COVID-19. Finally, the study involved questionnaires and follow-up visits by video call *via* WeChat in July 2021 to minimize the risk of COVID-19 infection (19, 20).

**Table 1:** Demographic data of participants.

**City**	**Number of medical staff(M / F)**	**Number of patiens** **(M / F)**	**Age of patiens**	**Number of patients with dental anxiety**	**Number of patients with contraindications**	**Number of patients with postoperative complications**
Chongqing	4(2/2)	36(9/27)	3−74	31	3	32
Beijing	3(1/2)	33(15/18)	5−68	23	2	27
Shanghai	3(2/1)	27(12/15)	4−55	18	1	26
Guangdong	2(0/2)	14(10/4)	6−48	10	1	13
Jiangsu	3(1/2)	22(6/16)	4−52	15	1	17
Henan	2(2/0)	15(8/7)	9−47	14	2	14
Shandong	2(2/0)	23(10/13)	6−53	14	1	18
Neimenggu	1(1/0)	10(6/4)	12−44	9	0	8
Total	20(11/9)	180(76/104)	3−74	134	11	155

### Applet description

**Figure 1:**
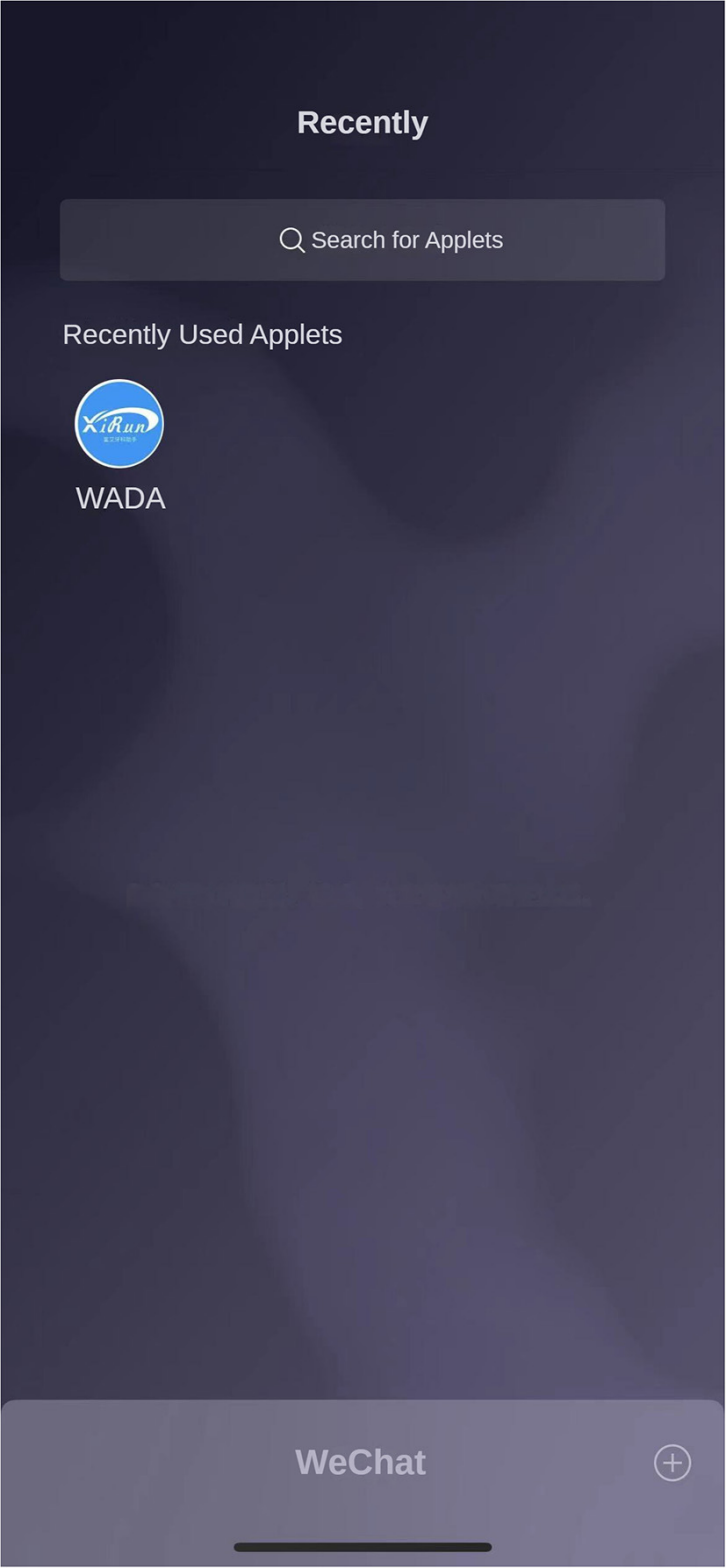
Applet opening interface. Patients can search for the WADA and use it by scrolling down on the main screen of WeChat without the need to download additional applications. It takes up little memory and is very convenient.

**Figure 2:**
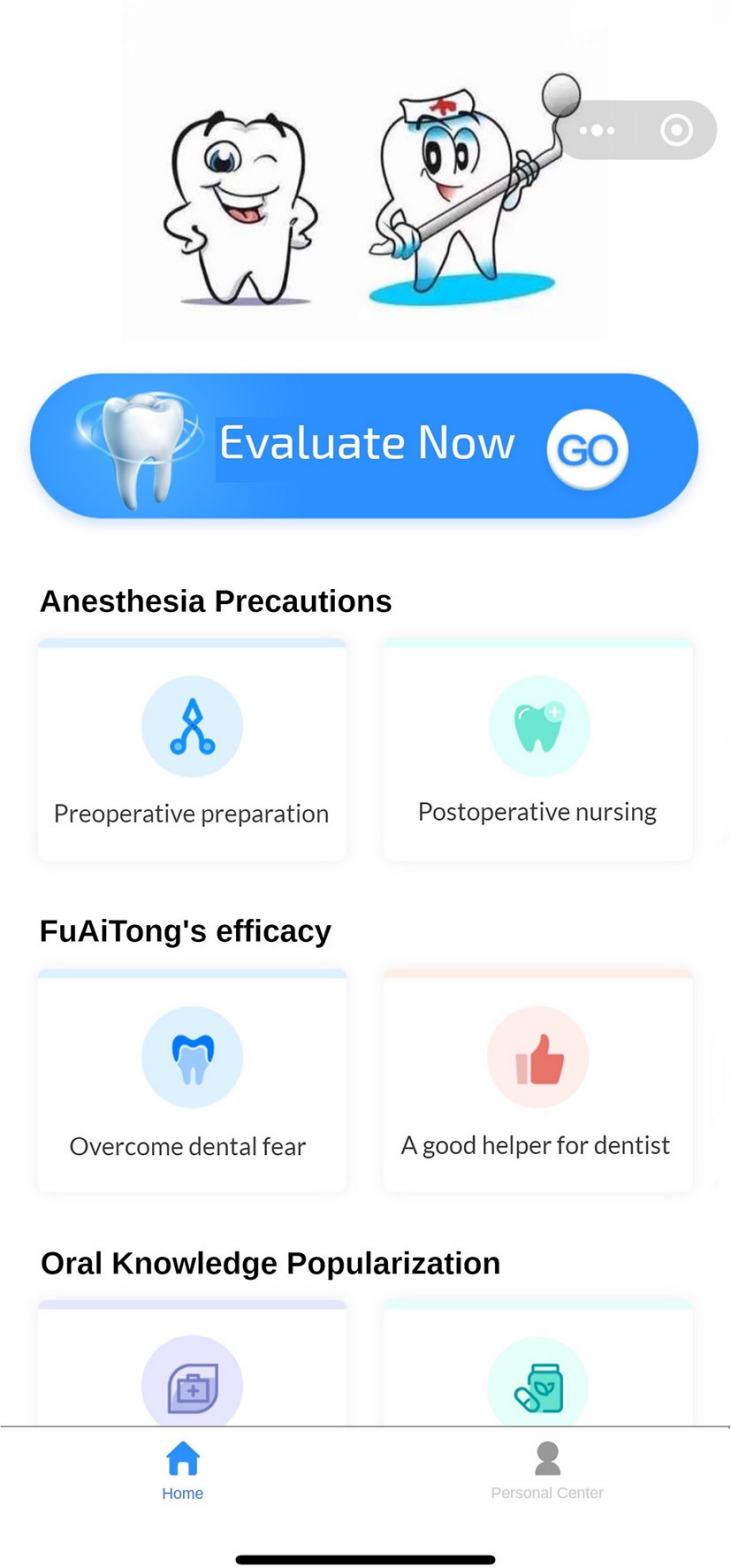
The main interface. It includes sections for immediate pre-treatment assessment, anesthesia instructions, dental knowledge, and personal center. Users can choose according to their needs.

**Figure 3:**
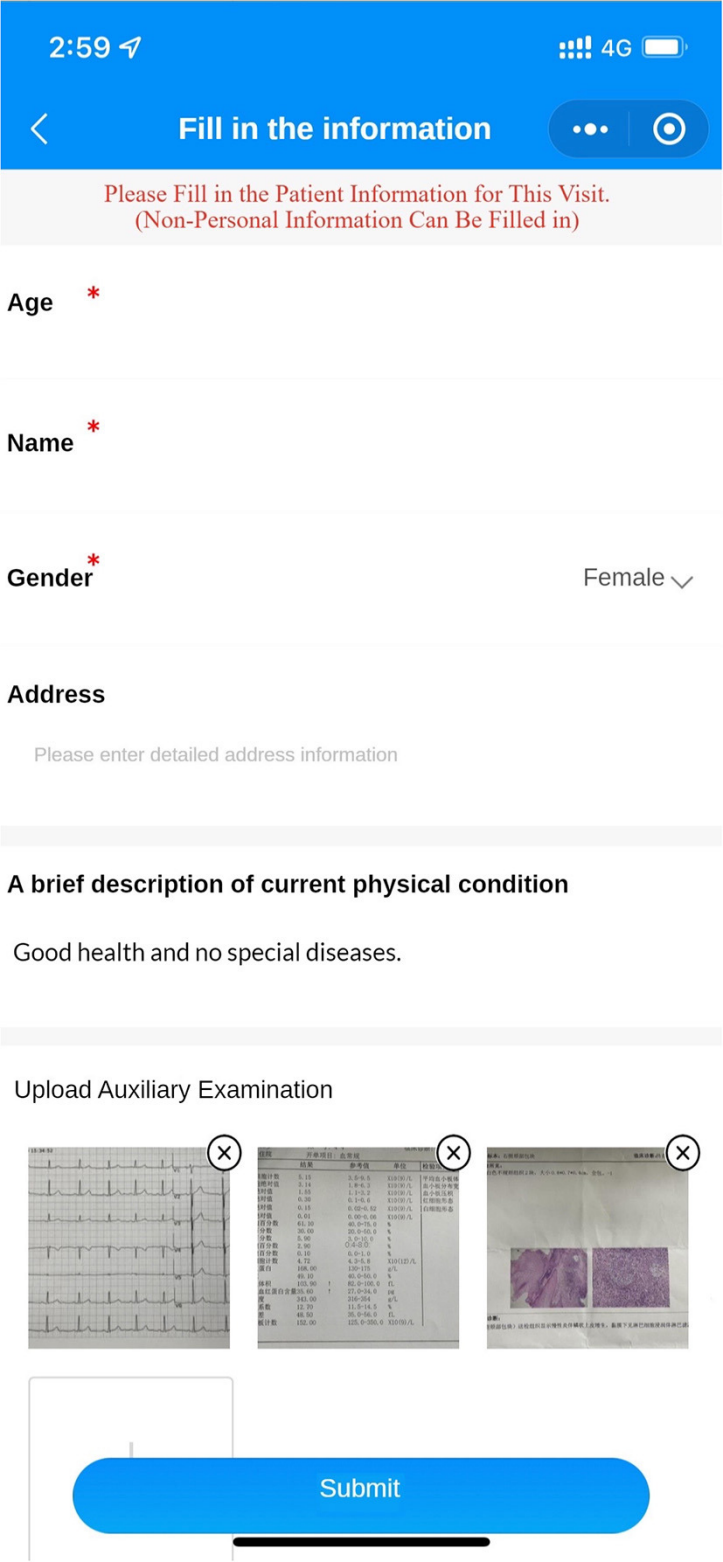
Personal information interface. Patients can fill in their personal information (name, sex and age) and their past medical conditions and upload examination results for the doctor to evaluate in advance.

**Figure 4:**
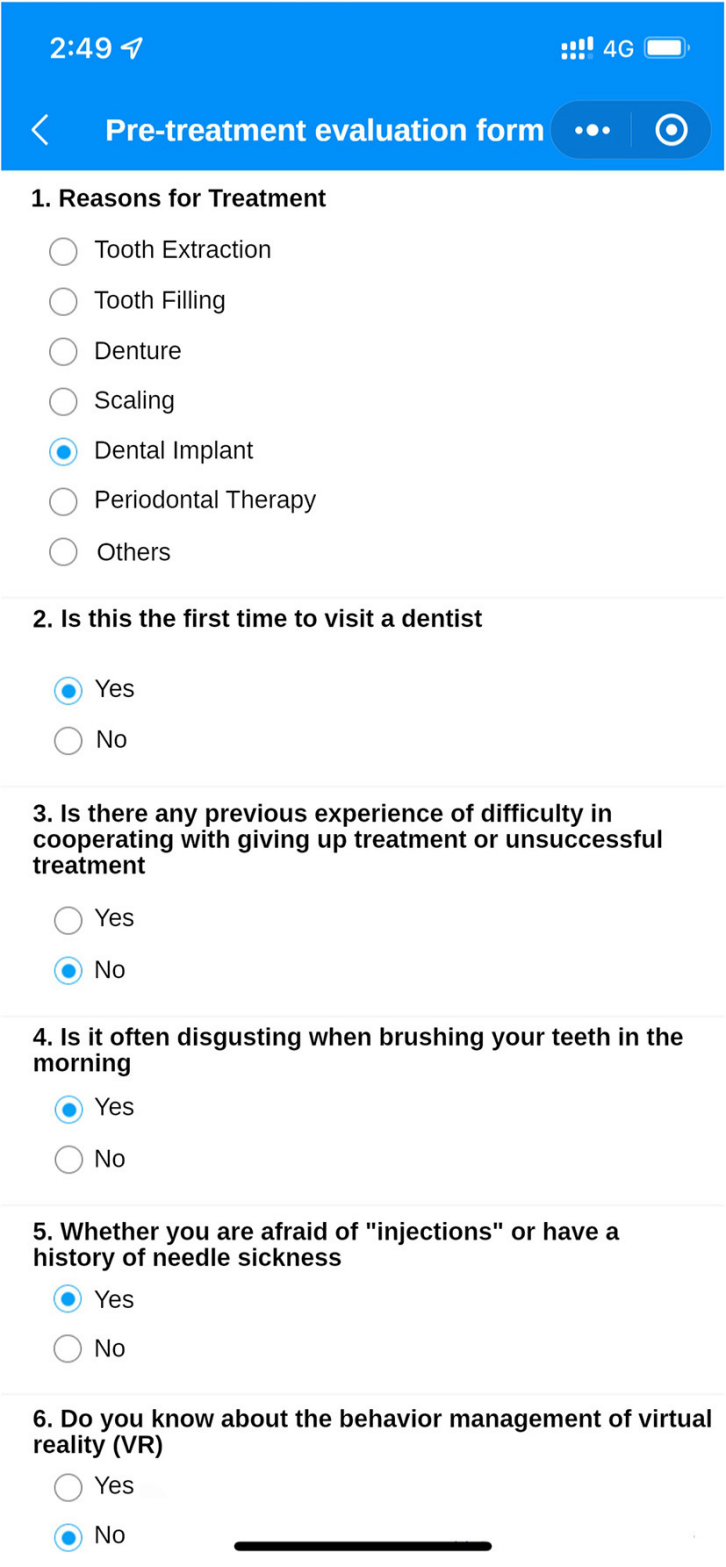
Pre-treatment assessment interface. Several questions are included, which the patient fills in according to their real situation. After filling in the questions, the intelligent assessment system will send the assessment results and risk prediction to the doctor's side.

**Figure 5:**
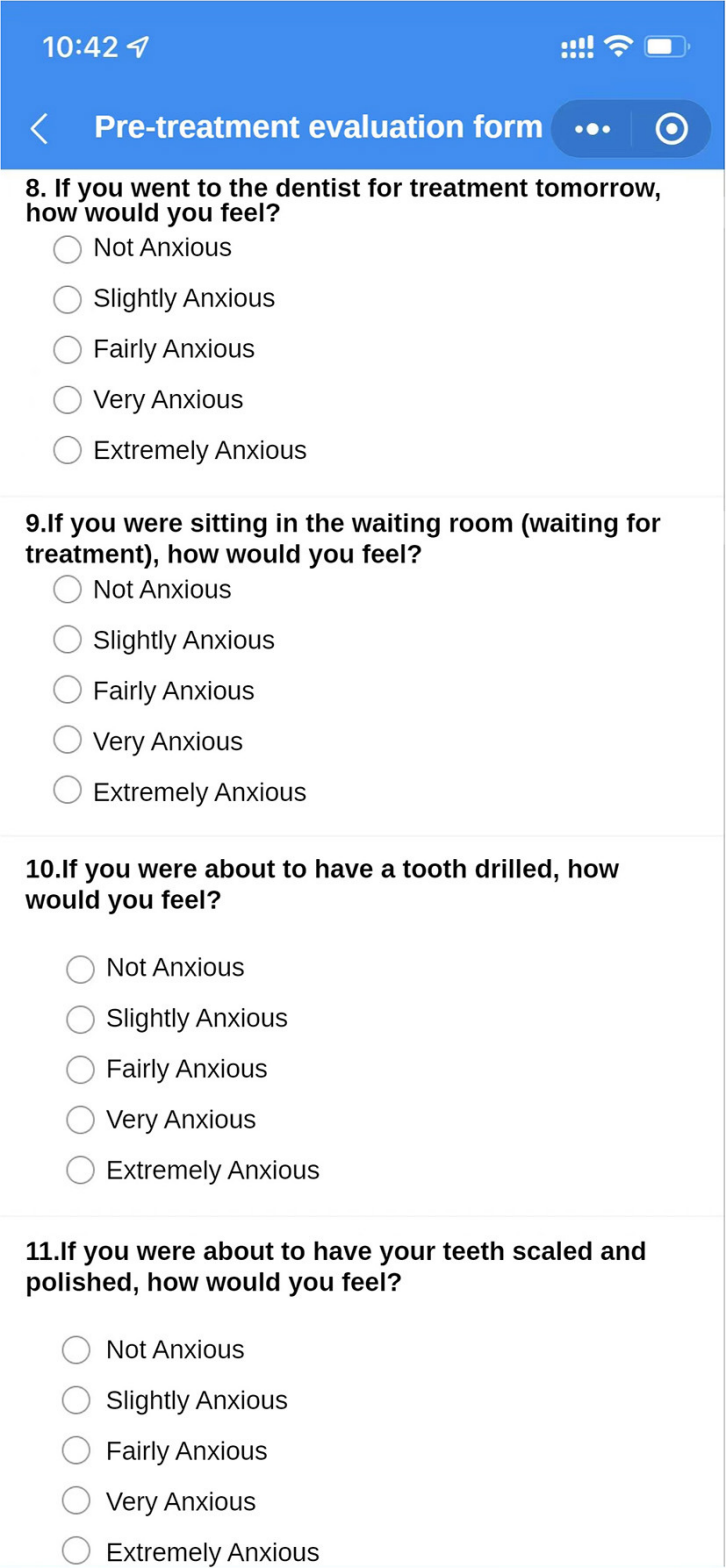
Pre-treatment assessment interface. Part of the Modified Dental Anxiety Scale (MDAS). After filling in the questions, the intelligent assessment system will send the assessment results and risk prediction to the doctor's side.

**Figure 6:**
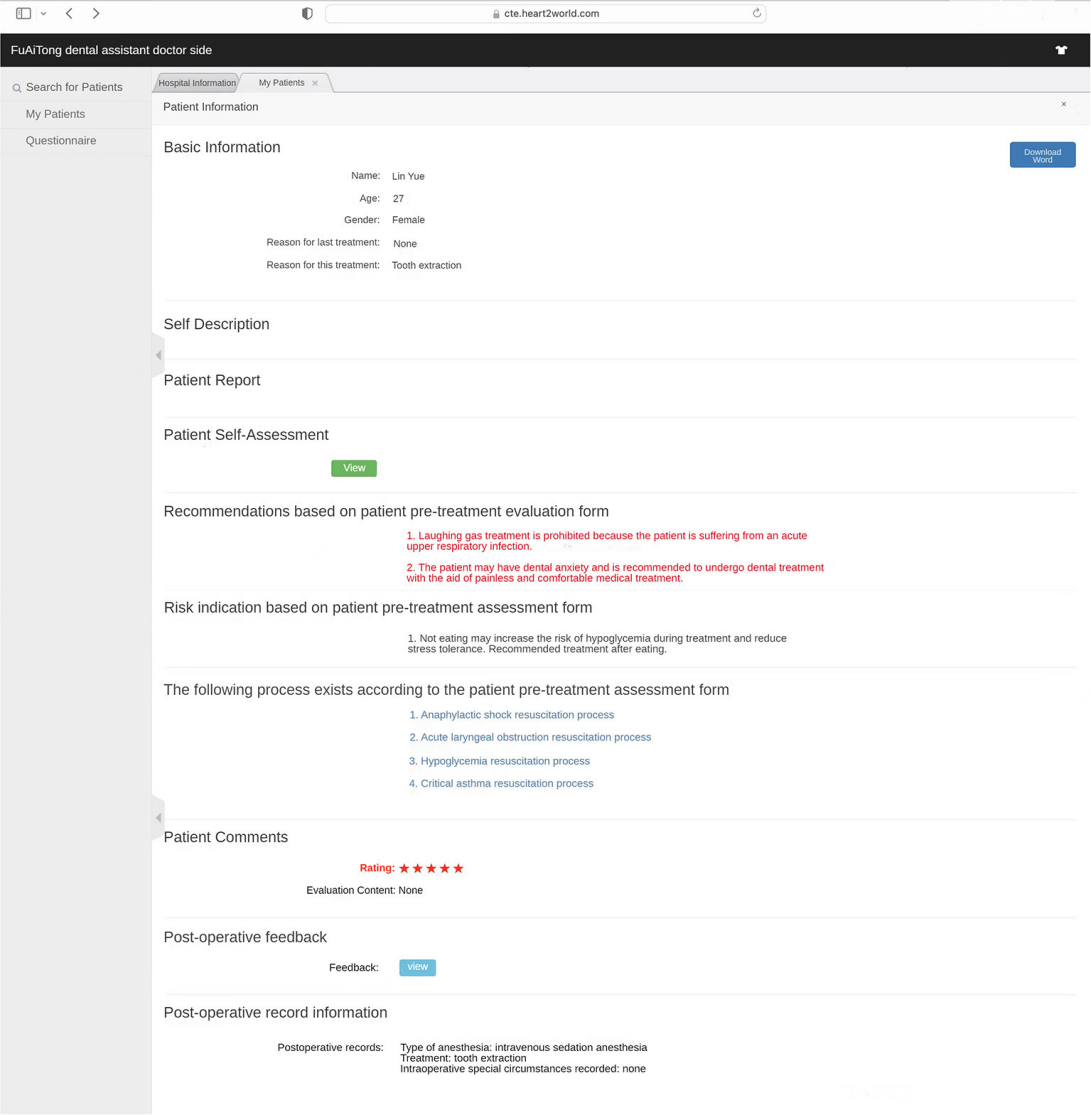
Doctor-side assessment result interface. Doctors can view patient assessment results, recommended anesthesia, risk warnings, post-operative records, etc. The symbol * means that the patient rated this treatment experience with a maximum of 5* and a minimum of 1*.

The WADA is a smartphone WeChat applet designed and developed by the Comfort Dentistry Center of the Stomatology Hospital affiliated with the Chongqing Medical University and has not been published anywhere. This is the first study on WADA. We incorporated the entire preoperative assessment system, including the Modified Dental Anxiety Scale (MDAS), Children Fear Survey Schedule-Dental Subscale (CFSS-DS), and various body systems assessment scales (Q4) such as allergy history, surgical history and various underlying diseases into the cloud database and WeChat applet through programming to establish logical relationships and perform intelligent assessments to assist anesthesiologists in decision-making. (Q1) Both MDAS and CFSS-DS are in Chinese and have been proven to be valid and reliable (21, 22). The applet realizes intelligent formulation of multi-level and multi-selective information fusion analysis to achieve personalized patient management. In turn, the collected big data can be used to perform intelligent assessment before treatment and help doctors to select different comfort treatment plans in a hierarchical manner through deep computer learning, finally realizing the closed-loop operation of the whole system and promoting the transformation of large-scale clinical data into medical knowledge. Using the internet, Internet of Things, cloud computing, and artificial intelligence technologies, we can realize smarter, safer, and more convenient medical services for both doctors and patients. As such, the WeChat-based applet needed three interfaces for the three relevant people: dentists, anesthesiologists, and patients, to improve the current dental medical consultation experience. The idea behind WADA is to allow patients suffering from dental anxiety to eliminate or alleviate their nervousness about dental visits by providing them general knowledge of dentistry and adequate pre-treatment assessment while minimizing the medical risks in dental treatment and reducing the repetitive and labor-intensive tasks of some dentists and anesthesiologists.

(Q5) WADA is a WeChat applet that anyone can start and use in WeChat at any time, without the need to download additional applications (Figure 1). There are many functional modules on the patient version of the WADA homepage (Figure 2), including personal center, pre-treatment assessment, pre-operative instructions, and dental knowledge. Users can access these modules after registration and login. In the Personal Center module, patients can fill in their basic information (name, sex and age) and upload the relevant examination results (Figure 3). At the same time, they can review the past visits in the visit record and give feedback on the last visit after treatment. (Q2) Through the pre-treatment assessment module, patients can make an CFSS-DS or MDAS and other intelligent assessment according to the age they filling in before treatment (Figures 4, 5), and then the system will automatically generate primary assessment results and provide medical advice to doctors. The preoperative information and dental science sections allow patients to browse preoperative preparation and postoperative care precautions, as well as to obtain oral health care knowledge. The doctors can use WADA to promptly see the patient's assessment results, as well as the treatment recommendations and risks based on the patient's pre-treatment assessment (Figure 6). When patients upload their questions or test results through the patient version of the app, the doctor is notified, and the patient is given instructions and medical advice through the doctor-facing homepage.

**Figure 7:**
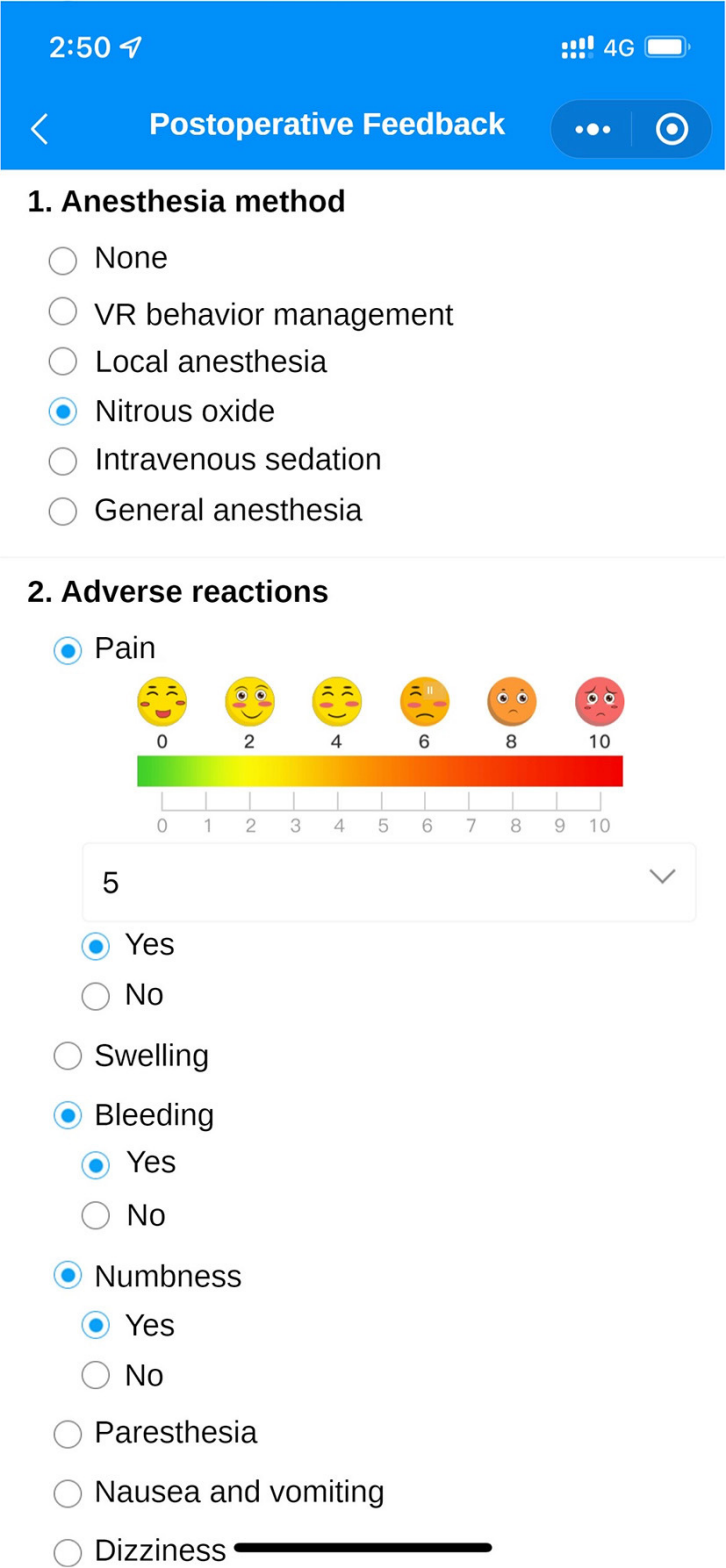
Post-treatment feedback interface. After the treatment, patients can fill in the feedback of this visit in the visit record, including the anesthesia method, whether there are any adverse reactions, etc. The same can be viewed by the doctor on the doctor's side.

The purpose of the application is:

1. Eliminate dental anxiety (through a patient-facing intelligent pre-treatment assessment platform system [Figures 4, 5] and general dental knowledge [Figure 2]).
2. Predict treatment risks and complications in advance (through a doctor-facing intelligent pre-treatment assessment results [Figure 6]).
3. Improve patient satisfaction (through post-treatment feedback system [Figures 3, 7]).
4. Reduce the work intensity of some anesthesiologists and reduce the risk of errors (through the intelligent pre-treatment assessment platform system on the doctor's side).
5. Collect data and knowledge for medical research.

### Data collection

During the 12-month survey period (August 2020 to July 2021), 200 participants had access to WADA at any time. They were instructed to use WADA before or after their dentist visit depending on their own needs but were not given any specific instructions on the frequency of usage. At the end of the survey period, 25 patients were asked to complete the SUS (Table 2) and undergo a usage experience interview (Table 3). The SUS is a simple, 5-point Likert scale that was developed as a fast and efficient method to collect an overview of the usability of a system (23, 24). SUS provides an overall usability assessment metric consisting of 10 questions, with odd-numbered questions being positive statements and even-numbered questions being negative statements. At the same time, 20 medical staff were interviewed about their usage experience (Table 3).

**Table 2:** The system usability scale.

	**Strongly disagree**.				**Strongly agree**
	**1**	**2**	**3**	**4**	**5**
1. I think that I would like to use this system frequently					
2. I found the system unnecessarily complex					
3. I thought the system was easy to use					
4. I think that I would need the support of a technical person to be able to use this system					
5. I found the various functions in this system were well integrated					
6. I thought there was too much inconsistency in this system					
7. I would imagine that most people would learn to use this system very quickly					
8. I found the system very cumbersome to use					
9. I felt very confident using the system					
10. I needed to learn a lot of things before I could get going with this system					
**There are ten questions on the SUS**. For problems with odd serial number, subtract 1 from their score. For problems with even serial numbers, the score is subtracted by 5. Add the final scores of all questions together and multiply by 2.5 and the calculated result is the SUS score of the applet. The range of score for each question is recorded as 0~4, the maximum score is 40, and the range of SUS score is 0~100. **Tips:** 1. Should not be summarized or discussed prior to completion. 2. Participants should be asked to complete each question quickly and without too much thought. 3.The second and sixth questions may be difficult for participants to understand and need to be explained clearly. 4. If the participant is unable to complete one of the questions for some reason, the participant is considered to have chosen the middle score for that question.					

**Table 3:** Examples from the semi-structured interview guide (*N* = 45).

**Sample questions of patient satisfaction**	**Sample questions of doctor effectiveness**
What is your immediate impression of this WeChat applet? Is it aesthetically pleasing?	What is your immediate impression of this WeChat applet? Is it aesthetically pleasing?
Which features do you use? And why?	Is this WeChat applet convenient and easy to use?
How would you rate the Intelligent Assessment System (is it relevant, easy to use and adequate)?	How would you rate the Intelligent Assessment System (is it relevant and accurate)?
How would you evaluate the dental knowledge section (is the information relevant and sufficient)?	Can this WeChat applet reduce the amount of repetitive work you have to do?
How do you assess the post-operative feedback function (is it relevant and how do you use it)?	Can this WeChat applet reduce the risk of dental treatment?
Is it possible to integrate this WeChat applet into your daily life? And how?	Is the anesthesia method recommended by the intelligent assessment system accurate?
What needs does the WeChat applet meet and what needs does it not meet?	What needs does the WeChat applet meet and what needs does it not meet?
What are the advantages and disadvantages of this WeChat applet?	What are the advantages and disadvantages of this WeChat applet?
Were you able to find the answers to the questions you were looking for?	

The return experience was guided by a semi-structured interview guide. The interview guide was driven by existing knowledge in the field and contained topics related to patient satisfaction, physician effectiveness, and multicenter experience (Table 4). The interviews involved open-ended and broad initial questions, which included the participants' direct impressions of WADA and considered new and unforeseen observations of the experience with opening questions followed by targeted follow-up questions.

**Table 4:** The usability assessment of the mobile system (*N* = 25).

	**Mean score**	**SD**
1. I think that I would like to use this system frequently	3.0	0.78
2. I found the system unnecessarily complex	2.90	0.70
3. I thought the system was easy to use	2.80	0.75
4. I think that I would need the support of a technical person to be able to use this system	2.90	0.83
5. I found the various functions in this system were well-integrated	2.70	0.90
6. I thought there was too much inconsistency in this system	3.20	0.75
7. I would imagine that most people would learn to use this system very quickly	2.70	0.90
8. I found the system very cumbersome to use	2.80	0.75
9. I felt very confident using the system	3.0	0.78
10. I needed to learn a lot of things before I could get going with this system	2.90	0.70
**The overall value of SUS**	72.25	

All interviews were conducted by video call *via* WeChat in July 2021. Interviews were conducted between one participant and one researcher. The first author (XLH) studied the relevant training and guidelines on the internet before conducting the interviews in Chinese. The interviews lasted for 45 min on average and were audio recorded and transcribed verbatim. Quotations were subsequently translated into English by a professional translator.

### Data analysis

The SUS scores were analyzed using the SPSS statistical software (version 17, SPSS, Inc., Chicago, IL, USA). Quantitative Likert scale data are presented as the mean ± standard deviation. According to Bangor and colleagues' thorough evaluation of the SUS, a system needs to score above 70 to be considered at least passable. Better systems score in the high 70s to high 80s, and scores over 90 indicate a truly superior system (25).

The interviews were recorded and transcribed verbatim. The Ritchie and Spencer qualitative Framework approach was used as the analytical framework. This involves: (1) familiarization with the data; (2) identification of a thematic framework; (3) indexation of the themes; (4) charting those themes into a hierarchical framework; and (5) mapping and interpretation of those themes (26). Using this analysis strategy, it was possible to mix theoretical and empirical perspectives, which made it applicable to both pre-existing knowledge and new unforeseen topics related to the participants' immediate experiences of using the applet.

To ensure consistency in the coding process, the authors independently performed steps 1 and 2, which involved familiarizing themselves with the richness and diversity of the data and making notes to develop ideas about the concepts and initial themes (identifying a thematic framework). After step 2, the authors met to discuss all concepts and initial themes that emerged from the transcripts to reach consensus on a set of related themes. The first author, XHu, then performed steps 3–5 to reorganize and annotate the data according to the thematic framework and to organize citations according to themes, which were found in the syndication process (indexing the themes). XHu then arranged all themes into a coded tree with related subthemes (charting themes into a hierarchical framework). Finally, XHu mapped and interpreted the dataset as a whole through a systematic process (mapping and interpretation) that culminated in a discussion among all authors to identify any overlooked insights.

Rigor was established to ensure dependability and credibility by properly transcribing data, individual analysis of data, discussing concepts and themes, and by validating the design through a theory-driven interview guide ensuring that all participants were asked the same range of questions.

## Results

During the survey period, 180 patients from eight different cities in China completed the survey. Of all respondents, 57.8% (104/180) identified as women and 42.2% (76/180) as men. The total SUS score of this WeChat applet was 72.25, which indicated good usability, learnability, and satisfaction.

The analysis of the participant interviews yielded a series of themes and sub-themes (Table 5).

**Table 5:** Themes and sub-themes.

**Patient satisfaction**	**Doctor effectiveness**	**Multi-center data integration**	**Increase the frequency of usage**
Eliminate tension and anxiety	Reduce repetitive work, effort, and risk	More comprehensive	
Increase doctor-patient communication and interaction	Remote diagnosis, advanced preparation, and detection of special cases		
Upload laboratory examination results	Optimize and improve treatment with previous visit results		
Give prompt feedback			

### Patient satisfaction

#### Eliminate tension and anxiety

Among the 180 patients who participated in the test, 134 were found to suffer from dental anxiety. WADA contains a library of popular science articles, including general knowledge about dentistry and oral health care from authoritative hospital-based dentists. Many participants knew the authors of these popular articles, which created a sense of authority and trustworthiness. Eighty-eight percent of the patients who participated in the interviews mentioned that the general knowledge and pre-treatment assessment before the visit made them feel more at ease.

“*WADA can help acquaint me with some oral-science information and provide treatment-related precautions, which can calm me and reduce my anxiety when facing the doctor and oral treatment.”* (18-year-old male participant from Beijing, China, who underwent tooth extraction).

#### Increase doctor-patient communication and interaction

Eighty-four percent of the patients who participated in an interview said that WADA has increased the interaction between them and their doctor and made their visit a more complete experience. They felt they could write their true feelings about the visit and upload the current examination results for the doctor to view in the post-treatment feedback.

#### Upload laboratory examination results

Fifty-six percent of the participants chose to take photos of their examination results and upload them before or after the treatment.

“*This way I can check the examination and treatment results after each visit and at any time without worrying about losing the data.”* (22-year-old female participant from Guangdong, China, who received a dental caries restoration)

In addition, the applet also helped saved time during the next visit.

“*When I visit a new hospital, I can access the results of previous examinations, which are readily available on WADA. This can help me save time and money needed to*

*undergo the examinations again.”* (38-year-old female participant from Chongqing, China, who underwent dental implant placement).

#### Give prompt feedback

Seventy-four percent of participants filled out a postoperative feedback form promptly after the treatment, which included questions regarding the type of anesthesia, adverse reactions, and any complications.

“*This allowed me to note how I felt in real time, for example, with regard to pain or other discomforts, and to communicate this with the doctor to avoid this experience during subsequent treatment.”* (36-year-old male participant from Chongqing, China, who underwent dental implant placement).

### Doctor effectiveness

#### Reduce repetitive work, effort, and risk

In today's aging and pediatric population, children with dental anxiety and elderly patients with multiple systemic complications are very challenging for dentists in their offices.

“*The applet helps reduce unnecessary repetition of steps and avoid errors in judgment due to excessive duplication of work, especially among the elderly people with many complications.”* (32-year-old anesthesiologist from Chongqing, China).

#### Remote diagnosis, advanced preparation, and detection of special cases

Four out of seven anesthesiologists mentioned that they could make an initial diagnosis remotely based on the pre-treatment assessment submitted by the patient and prepare medications and emergency measures in advance for possible complications.

“*For example, for patients with a history of a cardiac disease in the last 6 months, we can prepare emergency measures in advance. Patients with a history of hypertension can be instructed to take medication to lower their blood pressure and visit when the blood pressure normalizes, helping to save the time of both doctor and patient*.” (35-year-old anesthesiologist from Shangdong, China)

During the 12-month survey period, the WADA Smart Assessment System screened a total of 11 individuals with combined contraindications, 9 of whom were contraindicated for nitrous oxide anesthesia.

“*Patients are reminded that nitrous oxide therapy is prohibited when they have a history of intestinal obstruction, pneumothorax, or pulmonary fibrosis, which is very smart and reduces our duplication of workload”* (32-year-old anesthesiologist from Chongqing, China).

#### Optimize and improve treatment with previous visit results

Through the information that patients fill out in the postoperative feedback system at the end of the treatment, 86% of the patients had different degrees of complications such as pain, dizziness, and numbness. All anesthesiologists believe that the postoperative feedback function of WADA allowed them to reflect upon the treatment process.

“*We advise patients to complete the WADA post-operative feedback form following the treatment do that their feedback can be used for continuous adjustments and improvements to optimize the patient experience.”* (33-year-old anesthesiologist from Shanghai, China)

Three out of 7 dentists also mentioned the convenience of having patients' test results uploaded through WADA.

“*I habitually check the patients' previously uploaded test results when they come to the clinic, which saves time for both sides”* (36-year-old dentist from Henan, China).

### Multi-center data integration

#### More comprehensive data

WADA was utilized in multiple dental clinics of nine cities nationwide and was well accepted by dental professionals. This multi-center application allows easier dissemination of the results to a wider audience irrespective of regional differences and allows for continuous optimization of the intelligent assessment system and treatment modalities by analyzing patient data from each city.

### Increase the frequency of usage

As a WeChat applet, WADA uses simplified steps, and they can be opened directly without downloading the application package. When patients want to undergo self-assessment or receive a dental consultation at anytime and anywhere, they can open the relevant pages directly from the main WeChat interface, which makes the process convenient and can help increase the frequency of usage.

## Discussion

The traditional face-to-face medical consult model is no longer sufficient to meet the demand for medical services. Therefore, more and more medical institutions and computer scientists are using technologies to provide more effective and convenient medical services to patients. There are more than 318,000 medical apps available to help diagnose and manage diseases (27), for example, there apps for blood pressure monitoring (28) and electrocardiogram measurements (29).

Our PubMed search identified many articles on medical apps. These apps can be broadly classified into three categories according to the target user: patient-facing behavioral and health management apps (12, 30, 31), physician-facing apps to aid diagnosis (32–34), and apps to aid learning for medical students (35, 36). Regarding patient-facing apps, the most common categories are mobile health apps intended for monitoring and management of blood glucose levels (37–39) (e.g. SuCare, Sanofi-Aventis US LLC), blood pressure levels (39, 40), and cholesterol (41), with other apps for medical conditions (e.g., Vitadoc+, Medisana GmbH). Certain apps can alert patients when they need further professional help and others help to diagnose specific pathologies. In the dental field, in 2016, Francesca et al. found that oral hygiene compliance and reduced incidence of white spot lesions could be improved *via* the use of apps (42). In the same year, Janneke et al. launched an app called WhiteTeeth to improve oral hygiene in adolescents and determined its usability to be good using SUS (43). In 2020, Kim et al. identified the location and distribution of users' dental plaque *via* the hand-held LIF device or mobile app (44). Tobias et al. designed a mobile health app called iGAM, which uses selfies to monitor gingivitis (45), thereby facilitating the flow of information between the dentist and patient during the examination.

To our knowledge, there is no intelligent personalized assessment system for pre-treatment of common oral diseases in China or abroad. Therefore, we have designed and developed an intelligent preoperative evaluation platform system combined with artificial intelligence, moving the entire preoperative evaluation system to the cloud database and embedding it into a WeChat applet to establish logical relationships through programming to produce intelligent evaluation results to assist anesthesiologists in making decisions. In this study, we explored how participants evaluated the applet and its convenience, and the statistical results demonstrated the usefulness of WADA in reducing patient dental anxiety and increasing patient satisfaction.

On reviewing these existing dental applications (42–48), it was found that most of them monitor the patient's lifestyle and oral health status. None of them played a complete role in the whole process of patient treatment like WADA, including pre-treatment assessment, during-treatment advice, and post-treatment feedback.

The convenience provided by WADA points to four main areas: patient satisfaction, physician convenience, multi-center data integration, and warning of systemic risk in dental treatment. Systematic risk assessment becomes easier to perform along with dental treatment, and treatment complications can be predicted in advance and prevented, thereby improving the satisfaction and convenience experienced by both patients and doctor, enabling effective communication between patients and doctors, and allowing acquisition of oral health care knowledge. Overall, our survey showed that WADA is an intelligent and convenient medical application for dental treatment that contributes to the safety and efficiency of dental treatment. Patients also provided some key suggestions that will be considered in the subsequent development of the WADA upgrade.

Some references mention that filling a dental fear questionnaire before treatment may give false results as the child may experience anticipatory anxiety prior to treatment, which would be expressed through the questionnaire instead of the fear relating to the dental procedure in the moment. On the other hand, applying measures immediately after treatment might capture the child's dental fear, and children who have recovered after treatment may rate the treatment procedure more positively than their actual experience (2). Also, the duration of dental treatment was a factor in children's anxiety, which may be reflected in the study results (49). Therefore, future studies should attempt to standardize the assessment periods over the course of the treatment and follow-up (2).

### Limitations

Some limitations of this study should be considered. Patients who may have had an underlying anxiety disorder or a condition that may have affected their anxiety were not excluded. We also did not screen and exclude some factors that might influence the assessment of patients' dental anxiety disorders (e.g., self-concept, behavioral management, attention-deficit hyperactivity disorder, and oppositional defiant disorder) (50–53), which may have impacted the results.

According to the feedback provide by some, there is no way to provide real-time notifications for WADA due to the specificity of WeChat applets. Notifications would be a great help for people with busy lives. Another possible limitation is that the uneven distribution of males and females participating in this study created a response imbalance. In addition, the number of participants was uneven across age groups. Therefore, the results and feedback may have been different if the sex and age distributions were more equal.

It should also be noted that although this study covered multiple dental hospitals in multiple cities, it did not have full coverage across China. Future studies will be based on the final version of WADA and will be tested more comprehensively through efforts by all parties to recruit national beta users from all cities of China.

## Conclusion

In this study, we developed a pre-treatment intelligent personalized assessment system, which includes pre-treatment assessment, preoperative instructions, dental knowledge, post-treatment feedback on the patient side, and anesthesia modality recommendation and risk assessment by the doctor.

Overall, most participants were satisfied with the feedback on WADA. Patients and their caregivers were able to access more useful services through WADA. For some of the participants who were physicians, WADA provided a lot of convenience in providing treatment, especially through the post-treatment feedback and uploading of the test results. On the other hand, WADA also enhanced the convenience of doctors, saving time and reducing treatment risks, especially for high-risk patients during the COVID-19 pandemic.

## Data availability statement

The original contributions presented in the study are included in the article/supplementary files, further inquiries can be directed to the corresponding author/s.

## Ethics statement

The study was performed according to the World Medical Association's Declaration of Helsinki and the procedures were approved by the Ethics Committees of the Stomatological Hospital of the Chongqing Medical University (2020-023). All participants signed an informed consent form prior to participation. Use of anonymized data from the app for academic research purposes is allowed under the app's terms of service.

## Author contributions

CY, JZ, and XMH participated in the study concept and design. XLH collected the data and drafted the manuscript. XLH, JZ, NZ, and DJR participated in the analysis and interpretation of the data. JZ, CY, and LF critically revised the manuscript. All authors contributed to the article and approved the submitted version.

## Funding

The author(s) disclose receipt of the following financial support for the research, authorship, and publication of this article. Intelligent Medicine Project of Chongqing Medical University, China (Grant No: ZHYX202116); Engineering Research Center of Fujian University for Stomatological Biomaterials (Xiamen Medical College). (Grant No: XMMC-KQ202102).

## Conflict of interest

The authors declare that the research was conducted in the absence of any commercial or financial relationships that could be construed as a potential conflict of interest.

## Publisher's note

All claims expressed in this article are solely those of the authors and do not necessarily represent those of their affiliated organizations, or those of the publisher, the editors and the reviewers. Any product that may be evaluated in this article, or claim that may be made by its manufacturer, is not guaranteed or endorsed by the publisher.
